# Lifelong Memories That Help Persons Living with Dementia and Depression to Engage and Thrive

**DOI:** 10.1093/geroni/igaf122.3404

**Published:** 2025-12-31

**Authors:** Christina Miyawaki, Angela McClellan

**Affiliations:** University of Houston, Houston, Texas, United States; University of Houston, Houston, Texas, United States

## Abstract

Dementia and depression are common neuropsychiatric disorders in older Americans and 30% experience both conditions. Yet, interventions to address both disorders are scarce. Life review, a structured reminiscence in which people recall their lives chronologically, has demonstrated efficacy in depression. We developed a life review-based intervention, Caregiver-Provided Life Review (C-PLR), for people living with dementia and depression (PwDD) and their family caregivers. We trained caregivers in life review skills, and caregivers conducted life reviews with their PwDD at home. The purposes of this study were to report PwDD’s most pleasant and challenging life review sessions and how C-PLR can provide dyads with meaningful activities despite the disorders. Forty-five dyads completed the study. PwDD were 81 years old (mean), widowed, retired, female, and in poor/fair health. Caregivers were 58 years old, married, working, college-educated, female, and in good/excellent health. Dyads enjoyed the early childhood sessions because PwDD articulated their vivid long-term memories. However, older adulthood sessions were challenging because PwDD could not recall recent events highlighting their loss of short-term memories. Observing this progressive decline in memory across the intervention, however, is beneficial because caregivers can determine the extent of their PwDD’s decline, adjust their expectations, and plan their lives accordingly. With a lack of meaningful activities as a common unmet need in PwDD’s lives, C-PLR can provide PwDD meaningful opportunities to share their life stories from early childhood to the present. Thus, caregivers have a significant role to play for PwDD who wish to leave a legacy in their lives.

